# Systematic review and meta-analysis of type B aortic dissection involving the left subclavian artery with a Castor stent graft

**DOI:** 10.3389/fcvm.2022.1052094

**Published:** 2022-11-29

**Authors:** Shihua Yao, Xu Chen, Yalin Liao, Gangbing Ding, Dagang Li, Gengliang Qin, Ruiguo Qiao, Xin Sun, Qijun Zheng

**Affiliations:** ^1^Department of Cardiovascular Surgery, Shenzhen People’s Hospital, Second Clinical Medical College, Jinan University, Shenzhen, Guangdong, China; ^2^Department of Cardiovascular Medicine, Shenzhen People’s Hospital, Second Clinical Medical College, Jinan University, Shenzhen, Guangdong, China

**Keywords:** type B aortic dissection, single-branch stent graft, thoracic endovascular aortic repair, left subclavian artery, Castor stent graft

## Abstract

**Objective:**

Despite the rapid development of thoracic endovascular aortic repair (TEVAR), it is still a challenge to maintain the blood flow of the branch arteries above the aortic arch in Stanford type B aortic dissection involving the left subclavian artery (LSA). The Castor stent graft is an integrated, customized, single-branch stent that enables reconstruction of the LSA. The purpose of this systematic review and meta-analysis was to assess the efficacy of the Castor stent graft for type B aortic dissection.

**Materials and methods:**

An extensive electronic literature search (PROSPERO registration number: CRD42022322146) was undertaken to identify all articles published up to August 2022 that described thoracic aortic repair with branch stents in the treatment of type B aortic dissection involving the LSA. The quality of the included studies was analyzed using the MINORS criteria. The primary outcome measures were the technical success rate, early mortality rate, endoleak rate, and 1-year survival rate. The secondary outcome measures were the stroke rate, left upper extremity ischemia rate, and target vessel patency rate.

**Results:**

Eleven studies involving 415 patients were eligible for this meta-analysis. The LSA was successfully preserved in all procedures. The technical success rate was 97.5% (95% CI: 0.953–0.991); the intraoperative endoleak rate was 0.1% (95% CI: 0.000–0.012); the intraoperative LSA patency rate was 99.52%; the intraoperative LSA stent deformation and stenosis rate was 0.15% (95% CI: 0.000–0.051); the early type I endoleak rate was 1.6% (95% CI: 0.003–0.035); the 30-day mortality rate was 0.96%; the early reintervention rate was 0.9% (95% CI: 0.000–0.040); and the perioperative stroke rate was 0% (95% CI: 0.000–0.005). The 1-year survival rate was 99.7% (95% CI: 0.976–1.000). The half-year LSA patency rate was 99.3%, the 1-year LSA patency rate was 97.58%, and the 2-year LSA patency rate was 95.23%. During the follow-up period, the leakage rate was 0.3% (95% CI: 0.000–0.017), the incidence of left upper extremity ischemia rate was 0.5% (95% CI: 0.000–0.035), and the deformation and stenosis rate of the LSA stent was 2.2% (95% CI: 0.06–0.046).

**Conclusion:**

This meta-analysis shows that endovascular repair of type B aortic dissection using the Castor stent-graft may be technically feasible and effective. However, this conclusion needs to be interpreted with caution, as the quality of evidence for all outcomes is between low and very low.

**Systematic review registration:**

[https://www.crd.york.ac.uk/prospero/], identifier [CRD42022322146].

## Introduction

The International Registry of Acute Aortic Dissection (IRAD) (1) showed a 30-day mortality rate of 10% in patients with acute Stanford type B aortic dissection and a 25% mortality rate 3 years after the onset. TEVAR is currently the treatment of choice for TBAD, of which approximately 40% of TBAD involve the vicinity of the left subclavian artery orifice (2). Since, a segment of healthy aorta distal to the LSA may be lacking, in many cases, the subclavian artery coverage is necessary in order to obtain a suitable proximal landing zone (3). However, covering the LSA may lead to serious complications, such as spinal cord ischemia, intracranial ischemia, left upper limb ischemia, and type II endoleak (4). Therefore, for thoracic aortic dissection involving the LSA, whether to cover the LSA during TEVAR and how to reconstruct the LSA have always been controversial and heavily debated in the field of endovascular treatment of large vessels.

A castor stent graft (Microport Medical Co., Ltd., Shanghai, China) is a feasible customizable single-branch stent used in the reconstruction of the LSA during treatment for aortic dissection. It is the first unibody single-branched stent graft in China and has been approved by the China Food and Drug Administration since 2017 (5). The material of the Castor stent graft is a self-expanding woven polyester fabric, which has an integrated design in which the branch stent is sutured on the main stent (6). The main stent graft covers the primary entry tear of the aorta, and the branch stent graft proevents the LSA from being covered. The branch stent for LSA reconstruction has a length of 5–30 mm and can be moved backward, and the model of the branch stent can be selected individually (7). To ensure the blood supply of the left vertebral artery, the distal end of the branch stent should avoid covering the left vertebral artery. It was reported that more than 7,000 Castor branch stents were applied in the clinic in 2021 (8). In this review and meta-analysis, we analyzed the use of Castor branch stents in the treatment of Stanford type B aortic dissection, and evaluated its short- and medium-term clinical outcomes.

## Materials and methods

This systematic review and meta-analysis were performed in accordance with PRISMA guidelines and were registered on the PROSPERO website (Center for Reviews and Dissemination, University of York^1^) under registration number CRD42022322146.

### Literature source and search strategy

PubMed, Embase, Web of Science, CNKI, and WanFang Data were systematically searched for relevant articles that were published before August 2022 and reported on the outcomes of Castor stent grafts. Our search terms were: (“DeBakey type III aortic dissection” OR “type B aortic dissection”) AND (“endovascular” OR “endograft”) AND (“stent graft” OR “single branch” OR “Castor”). In addition, the reference lists of all retrieved articles were examined for further relevant series.

### Selection criteria

Two authors (Yao and Chen) independently conducted the literature search. The titles and abstracts of all citations were independently reviewed to identify potentially relevant studies and exclude any duplications. The full text of the corresponding reports was reviewed to assess whether the studies met the inclusion and exclusion criteria. The references within these articles were also analyzed. The inclusion criteria included the following: (I) Described the application of Castor stent grafts in the treatment of Stanford type B aortic dissection; (II) Provided baseline characteristics of the recruited patients; (III) Reported on a series of at least 8 patients to prevent bias arising from small sample populations; (IV) Had a postoperative follow-up time ≥ 3 months. The exclusion criteria included the following: (I) Type of study that included *in vitro* experiments and animal experiments; (II) Diseases studied that included Stanford type A aortic dissection, aortic aneurysm, intramural hematoma, or aortic ulcer; (III) Studies that used other techniques, such as chimney, fenestration or hybrid, to reconstruct the LSA; (IV) When patients from the same center were reported repeatedly, only those studies with larger sample size, and longer follow-up, or the more recently reported studies were selected.

### Definitions and data extraction

The preliminary data extraction was completed independently by two staff members, who discussed and negotiated any data with discrepancies. The definition of the main statistical variables was as follows: ① Technical success rate: the stent graft was successfully deployed, covering the primary entry tear while preserving the LSA, and there was no type I endoleak at the end of the operation; ② Early endoleak: there was flow between the stent and the aortic wall during the operation and within 30 days after the operation, including type I endoleaks that were resolved with immediate adjuvant measures during the operation and intraoperative angiography and examination within 30 days after the operation; ③ 30-day mortality: mortality during surgery and within 30 days after surgery; ④ Early reintervention: the number of treatments that were repeated during surgery and within 30 days after surgery to remedy aortic-related disease; ⑤ Perioperative stroke incidence: the probability of developing cerebral ischemic symptoms during the perioperative period; ⑥ LSA patency rate: the probability that LSA has no occlusion and normal blood flow; and ⑦ NA: these data are not explicitly stated in the article or are missing from the article.

### Quality assessment

The quality of the articles was assessed by 2 authors using the methodology index for non-randomized studies (MINORS) (9). Any disagreements by the authors during the literature search, literature selection, quality assessment, or data extraction process were resolved by consensus. If an agreement was reached, a third party was consulted, and a final decision was made after reaching a consensus.

### Data synthesis and heterogeneity assessment

Descriptive statistics were used to assess the patients’ baseline characteristics, aortic dissection characteristics, anesthesia modality, and device characteristics. Continuous variables are described as the mean ± standard deviation. When a variable was calculated to contain NA, the data points containing NA were excluded from the calculation of this variable. The meta-analysis was performed using the “metaprop” routine in Stata version 15 for Windows (10), which requires the Freeman-Tukey double arcsine transformation process and the DerSimonian-Laird random effects model (11). First, the Freeman-Tukey double-arcsine transformation process stabilizes the variance between studies, and then the DerSimonian-Laird random-effects model calculates weighted global pooled estimates. Forest plot graphs were used to illustrate the weighted outcomes as well as the pooled estimation with the 95% confidence interval (CI). Finally, publication bias was assessed by funnel plots. The heterogeneity among studies was tested by Thompson’s I^2^ statistics, with *I*^2^ > 50% and a *P* value ≤ 0.1 indicated significant heterogeneity (12). Random-effects models were used for data with significant heterogeneity, otherwise Fixed-effects analysis was used.

### Grading of evidence

The overall certainty of the body evidence was evaluated using the Grading of Recommendations Assessment, Development and Evaluation (GRADE) approach. By this tool, the evidence can be graded as being of high, moderate, low, or very low quality; however, evidence derived from observational studies receive an initial grade of low quality.

Criteria that downrates the quality of evidence include risk of bias (low MINORS score), imprecision (the 95% CI for effect estimates are wide or cross a minimally important difference of 10% for benefit or harm), inconsistency (substantial unexplained interstudy heterogeneity, *I*^2^ > 50%), indirectness (presence of factors that limit the generalizability of the results), and publication bias (evidence of small-study effects). The grade for observational analyses can be improved by a large magnitude of effect, the dose-response gradient, or attenuation by plausible confounding (13–18).

## Result

### Study selection and quality assessment

**FIGURE 1:**
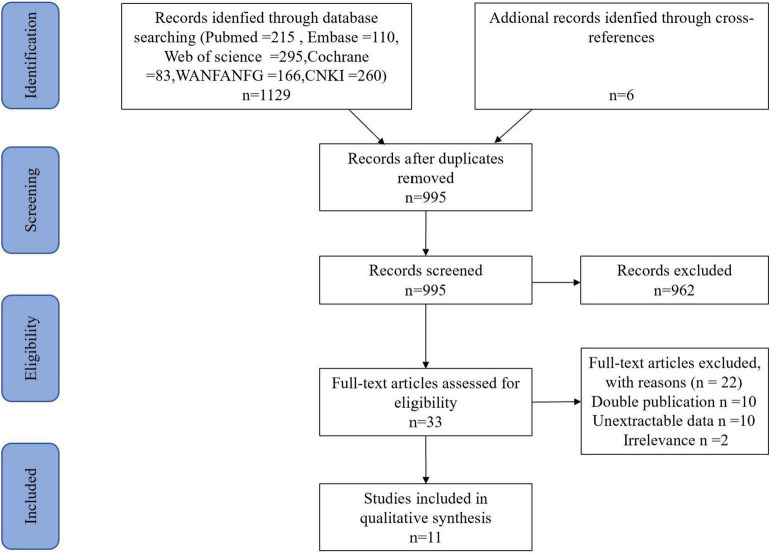
Study flow diagram.

According to the search keywords, a total of 1,129 records were found, and another six records were found according to the references, similar literature and related links. Ultimately, a total of 1,135 records were retrieved, and after excluding 134 duplicate records, 995 records remained. According to the inclusion and exclusion criteria, 33 articles met the requirements, and the full text of each article was obtained for reading. Ten articles with duplicate data, 10 articles with inseparable data, and 2 articles irrelevant to aortic dissection were excluded, and 11 articles were finally screened out. The excluded literature and the reasons for its exclusion are shown in Supplementary Table 1. Figure 1 is a flow chart showing the article selection process.

**FIGURE 2:**
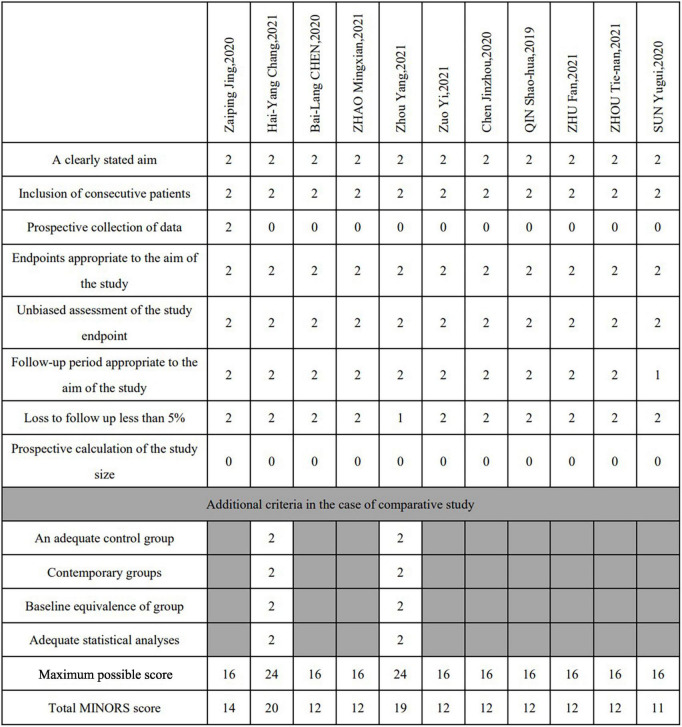
Study quality assessment (MINORS score). For noncomparative studies the quality was considered poor at a score ≤8, moderate at 9–14, and good at 15–16. The cutoff points were ≤14, 15–22 and 23–24, respectively, for comparative studies.

Regarding the study design, 1 study (5) was a multicenter prospective trial, 10 studies (19–28) were retrospective designs, and 2 studies (19, 22) were comparative studies. The quality of the included literature was assessed using the MINORS criteria, and the results showed that 11 studies were of moderate quality. There was no disagreement among the authors on the inclusion and exclusion criteria of the studies and the quality assessment of the studies. The process for assessing the quality of the literature quality assessment process is shown in Figure 2.

### Baseline patient characteristics

Eleven studies involving a total of 415 patients from 23 large medical centers were included in the meta-analysis, of which 27.86% of the patients used products premarketing and 72.14% used products post marketing. The patient enrolment period was from April 2013 to July 2021. The average age of the patients was 73.85 years, 69.9% of the patients were male, and the average follow-up time was 19.89 months. The basic characteristics of the patients are listed in Table 1.

**TABLE 1:** Baseline patient characteristics and details of aortic dissection.

References	Study period	Sample size	Male	Age (y)	Follow-up months	ATBAD	CTBAD	Details of aortic dissection			
								Primary ET to LSA distance (mm)	AD to LCCA distance (mm)	LSA diameter (mm)	LSA to LCCA distance (mm)
Jing et al. (5)	2013.04–2015.03	73	55	56.81 ± 13.30	61	50	23	12.43 ± 7.66	24.43 ± 8.73	8.92 ± 1.32	NA
Chang et al. (19)	2016.01–2019.12	31	NA	56.50 ± 10.80	15.19 ± 2.68	31	0	NA	NA	NA	NA
Chen et al. (20)	2017.06–2019.09	12	10	55 ± 14.9	24	NA	NA	< 15 mm	NA	11.75	14.25
Zhao et al. (21)	2016.01–2019.12	122	63	66.23 ± 9.87	12	65	57	< 15 mm	NA	NA	NA
Zhou et al. (22)	2018.03–2019.03	33	25	58.17 ± 10.96	7.23 ± 1.99	NA	NA	< 15 mm	NA	NA	NA
Zuo et al. (23)	2020.01–2021.07	31	26	55.5 ± 11.6	9	25	6	NA	30.3 ± 13.3	9.2 ± 1.5	9.65 ± 4.58
Chen et al. (24)	2017.07–2018.10	19	15	52.6 ± 11.7	6	19	0	7.8 ± 5.8	NA	12.5 ± 5.5	12.1 ± 2.7
Qin et al. (25)	2017.10–2018.06	18	11	64.4 ± 15.3	8 ± 2	17	1	< 15 mm	NA	9.23	7.69
Zhu et al. (26)	2019.04–2020.01	8	7	42	7.4	NA	NA	< 15 mm	NA	NA	11.75
Zhou et al. (27)	2018.05–2021.07	10	8	59.4 ± 13.76	21.64 ± 5.61	NA	NA	≤ 5 mm	8.20 ± 1.15	9.67 ± 1.01	8.2 ± 1.15
Sun et al. (28)	2018.08–2019.08	58	46	57.1 ± 13.2	3	41	4	< 15 mm	NA	9.5 ± 1.8	9.9 ± 3.1
Jing et al. (5)	2013.04–2015.03	73	55	56.81 ± 13.30	61	50	23	12.43 ± 7.66	24.43 ± 8.73	8.92 ± 1.32	NA
Chang et al. (19)	2016.01–2019.12	31	NA	56.50 ± 10.80	15.19 ± 2.68	31	0	NA	NA	NA	NA

*ATBAD, Acute type B aortic dissection; **CTBAD, Chronic type B aortic dissection; ***Primary ET to LSA distance: the distance between LSA and the primary entry tear.

The proximal anchoring zone refers to the distance between the LSA and the primary entry tear. It is generally considered that a distance ≥ 15 mm is ideal (29). If it is less than 15 mm, the anchoring effect of the proximal stent will be seriously affected (30). Across the 11 studies, the mean healthy anchoring area of the aorta was less than 15 mm in all patients, with one study reporting a healthy anchoring area of less than 5 mm in 10 patients. The characteristics of the aortic dissections are shown in Table 1.

### Perioperative outcomes

**FIGURE 3:**
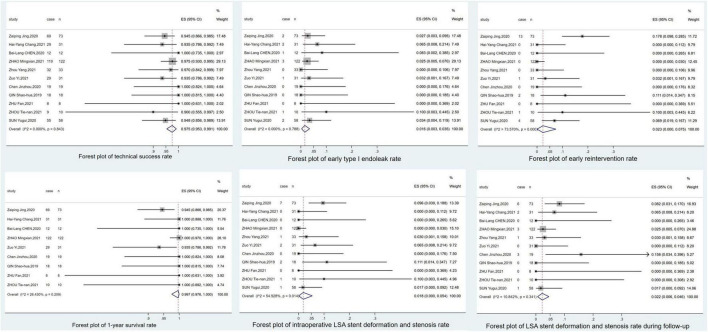
Forest plots.

A total of 415 patients in 11 studies were treated with Castor stent grafts. The details of the use of Castor stent grafts are shown in Table 2. Surgery was performed in 70.5% (248/352) of the patients in the acute presentation period and 29.5% (104/352) of the patients in the chronic presentation period, with an average operation time of 122.25 min; 73.7% (160/217) of the patients had general anesthesia, 11.1% (24/217) had local anesthesia, and 0.9% (2/217) had spinal anesthesia. The technical success rate was 97.5% (95% CI: 0.953–0.991). The intraoperative endoleak rate was 0.1% (95% CI: 0.000–0.012) and the early type I endoleak rate was 1.6% (95% CI: 0.003–0.035). The 30-day mortality rate was 0.96% (4/415), the early reintervention rate was 2.3% (95% CI: 0.000–0.075), and the perioperative stroke rate was 0% (95% CI: 0.000–0.005). There were no cases of paraplegia and 2 cases of stroke during the perioperative period. There was significant heterogeneity in the analysis of early reintervention rates (*I*^2^ = 73.6%>50%, *P* < 0.001). A sensitivity analysis of the 11 included studies showed that “Jing, (5)” had the greatest impact on heterogeneity. After removing this study and testing for heterogeneity in the remaining 10 studies, the early reintervention rate was 0.9% (95% CI: 0.000–0.040, *I*^2^ = 45.8% < 50%, *P* = 5.5%), suggesting that there was no significant heterogeneity, so the “Jing, (5)” should be removed from the analysis of “early reintervention rate”. The sensitivity analysis plot is shown in the Supplementary Figure 1. The details of the perioperative period are shown in Table 2. The forest plots are shown in Figure 3.

**TABLE 2:** Perioperative outcomes and details of the use of Castor branch stents.

References	Perioperative outcomes							Details of the use of Castor branch stents					
	Technical success rate	Surgery time (min)	30-day deaths	Early type I endoleak rate	Perioperative stroke	Intraoperative endoleak	Early reinter- vention	Diameter of proximal anchoring zone (mm)	Proximal anchoring zone length (mm)	Proximal diameter of branch stent (mm)	Proximal diameter of main stent (mm)	Average length of main bracket (mm)	Oversize rate of aortic landing zone proximal to main stent
Jing et al. (5)	69/73 (94.5%)	128.23 ± 66.83	2	2	0	1	13	30.66 ± 2.94	12.1 ± 1.8	10.11 ± 0.99	32.55 ± 2.61	165.4 ± 32.6	5.4% ± 3.1%
Chang et al. (19)	29/31 (93.5%)	149.23 ± 34.69	0	2	0	1	0	34.31 ± 3.18	5.39 ± 2.91	NA	NA	NA	NA
Chen et al. (20)	12/12 (100%)	NA	0	1	0	0	0	30.83	NA	10 12	NA	NA	10 15%
Zhao et al. (21)	119/122 (97.5%)	NA	0	3	1	0	0	NA	NA	NA	NA	NA	NA
Zhou et al. (22)	32/33 (97.0%)	136.0 ± 40.3	0	0	0	0	0	NA	NA	NA	NA	NA	NA
Zuo et al. (23)	29/31 (93.5%)	151.8 ± 48.5	2	1	1	1	1	31.8 ± 3.6	NA	10.3 ± 1.3	33.2 ± 3.5	NA	NA
Chen et al. (24)	19/19 (100%)	103.3 ± 15.2	0	0	0	0	0	30.4 ± 3.6	NA	10.14	32 40	NA	5 15%
Qin et al. (25)	18/18 (100%)	127.8 ± 20.1	0	0	0	0	2	NA	NA	NA	32 38	180–200	0 10%
Zhu et al. (26)	8/8 (100%)	47.5 ± 10	0	0	0	0	0	NA	NA	NA	NA	NA	NA
Zhou et al. (27)	9/10 (90%)	NA	0	1	0	0	1	NA	NA	6 12	28 36	200	10∼20%
Sun et al. (28)	55/58 (94.8%)	91.5 ± 26.4	0	2	0	2	4	30.5 ± 2.1	13.2 ± 1.3	NA	NA	NA	NA

### Follow-up outcomes

**FIGURE 4:**
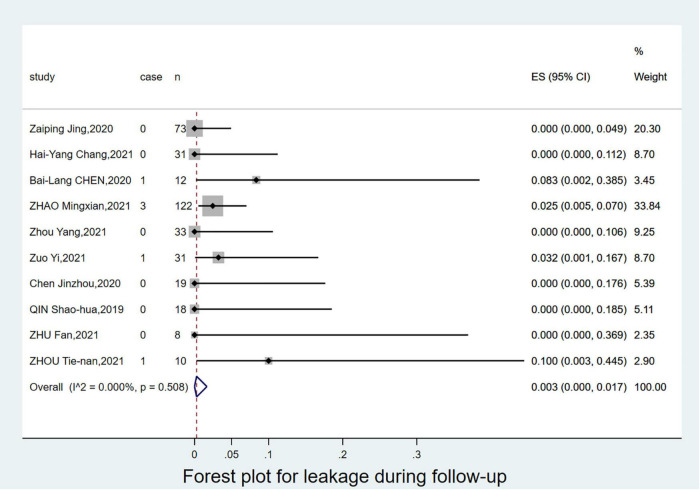
Forest plot for leakage during follow-up.

The 1-year survival rate was 99.7% (95% CI: 0.976–1.000; Figure 3), and the leakage rate during the follow-up period was 0.3% (95% CI: 0.000–0.017; Figure 4). During the follow-up period, there was 1 case of stroke, no cases of paraplegia, 2 retrograde type A dissections, and 2 cases of left brachial artery thrombosis. The complication rate during follow-up was 8.6% (95% CI: 0.039–0.146). Details of the follow-up outcomes are shown in Supplementary Table 2.

### Left subclavian artery patency

During surgery, 10 additional LSA stents were implanted. Among them, nine were implanted in patients with stenosis due to distortion of the LSA stent; the blood flow in the LSA was smooth after balloon stent or bare stent expansion. There were two cases of complete LSA occlusion due to failure of LSA stent release; no treatment was performed during the operation, and there were no left upper limb ischemia symptoms after the operation. There were no cases of left upper limb ischemia during the perioperative period. Therefore, the intraoperative LSA patency rate was 99.52% (413/415). The intraoperative LSA stent deformation and stenosis rate was 0.15% (95% CI: 0.000–0.051). During the follow-up period, there were nine patients with LSA occlusion, including two cases of intraoperative LSA occlusion and seven cases of new LSA occlusion during the follow-up period, but three cases of left upper limb ischemia due to stent stenosis. During the follow-up period, the deformation and stenosis rate of the LSA stent was 2.2% (95% CI: 0.06–0.046). The LSA patency rate at half of the year after the operation was 99.3%, at 1 year after the operation was 97.6%, and the LSA patency rate at 2 years after the operation was 95.2%. One patient had left brachial artery thrombosis, and the ischemic symptoms were alleviated after anticoagulation treatment. The other three patients had poor root deployment of the branch stent, and the ischemic symptoms were relieved after balloon stent expansion. Therefore, there were four patients with left upper limb ischemia during the follow-up period and the incidence of left upper extremity ischemia during follow-up was 0.5% (95% CI: 0.000–0.035). The complications of LSA stents and LSA patency are shown in Table 3. The forest plots are shown in Figure 3.

**TABLE 3:** Complications of left subclavian artery (LSA) Stents and left subclavian artery (LSA) patency.

References	Intraoperative complications of LSA					Complications of LSA during follow-up					LSA patency rate		
	LSA occlusion	LSA twist and stenosis	Causes of LSA stenosis and occlusion	Additional LSA stent implan- tation	Left upper extremity ischemia	LSA occlusion	LSA twist and stenosis	Causes of LSA stenosis and occlusion	Additional LSA stent implan- tation	Left upper extremity ischemia	6 months	1 year	2 years
Jing et al. (5)	1	6	1 case of LSA stent did not fully enter LSA, LSA caused occlusion; 6 cases of LSA stent distorted stenosis	6 stents	0	6	0	1 case of LSA stent was occluded during operation and was not treated during operation. During the follow-up period, 5 new cases of LSA occlusion occurred.	0	0	98.6%	97.3%	91.8%
Chang et al. (19)	0	0	—	0	0	2	0	2 cases of new LSA occlusion of unknown cause	0	0	100%	96.8%	93.5%
Chen et al. (20)	0	0	—	0	0	0	0	—	0	0	100%	100%	100%
Zhao et al. (21)	0	0	—	0	0	0	3	3 cases of new-onset LSA stent deformation	0	0	99.2%	97.5%	—
Zhou et al. (22)	0	1	1 case of LSA stent torsion and stenosis	0	0	0	1	1 case of unexplained new LSA occlusion	0	0	100%	—	—
Zuo et al. (23)	0	2	2 cases of LSA stent not fully pulled into LSA, and LSA stent was twisted and folded	0	0	0	0	—	0	0	100%	—	—
Chen et al. (24)	0	0	—	0	0	0	3	3 cases of poor root deployment of LSA stent.	3 balloon stents	4	100%	—	—
Qin et al. (25)	0	2	2 Cases of LSA stent torsion and stenosis	2 balloon stents	0	0	0	—	0	0	100%	—	—
Zhu et al. (26)	0	0	—	0	0	0	0	—	0	0	100%	—	—
Zhou et al. (27)	0	1	1 case of poor expansion of LSA stent	1 balloon stent and 1 bare stent	0	0	0	—	0	0	100%	100%	100%
Sun et al. (28)	1	0	1 case of LSA occlusion was caused by difficulty in releasing the LSA stent.	0	0	1	0	1 case of intraoperative occlusion, which was not treated intraoperatively	0	0	98.3%	—	—
Total	2	12	3 cases of LSA stents were difficult to release; 8 cases of LSA stents were twisted.	10 additional LSA stents	0	9	7	2 cases of intraoperative occlusion, 7 cases of new LSA occlusion, 7 cases of new LSA stent distortion	3 balloon stents	4	99.3%	97.6%	95.2%

*LSA patency: refers to the presence of blood flow signals in the LSA on imaging. **LSA occlusion: Refers to the complete occlusion of the LSA on imaging.

### Result of grading of recommendations assessment, development, and evaluation assessment

Although no factors warranting a downgrade were identified, the overall certainty of the body evidence was graded as low to very low, which was consistent with the default level for observational studies. Details of the GRADE assessment are shown in Supplementary Table 3.

## Discussion

### The importance of intraoperative reconstruction of the left subclavian artery in thoracic endovascular aortic repair

Numerous studies have shown that covering the LSA during surgery increases the risk of spinal cord ischemia and postoperative stroke. A meta-analysis conducted by Rizvi (31) showed that TEVAR covered with LSA was associated with 6% of patients having upper extremity ischemia, 4% having spinal cord ischemia, 2% having basilar artery ischemia, and 2% having anterior circulation cerebral infarction and a 6% death rate. Another study suggested that patients who received LSA coverage during TEVAR, followed by additional revascularization, could have a reduced incidence of spinal cord ischemia (32). Especially for some patients whose left vertebral artery is open to the LSA, covering the LSA may lead to paraplegia in patients with right vertebral artery absence due to spinal cord ischemia. It is stated in the American Society of Vascular Surgery practice guidelines (33) that the LSA should be reconstructed during TEVAR procedures involving the LSA, especially for the absence of atresia or occlusion of the right vertebral artery, the termination of the left vertebral artery in the posterior inferior cerebellar artery, after coronary artery bypass grafting, left arm dialysis arteriovenous, etc. To avoid serious complications caused by covering the LSA, the LSA should be reconstructed during TEVAR. Currently, the techniques that can performed to reconstruct the LSA include chimney technology, hybrid techniques, fenestration techniques, branch stent techniques, etc.

In the current systematic review, we provide contemporary and comprehensive technical data detailing the perioperative and interim results of the Castor stent graft technique. The data of this meta-analysis showed that the technical success rate was 97.5%, the 30-day mortality rate was 0.96%, the early type I endoleak rate was 1.6%, the intraoperative LSA patency rate was 99.52%, the intraoperative LSA stent deformation and stenosis rate was 0.15%, and the perioperative stroke rate was 0%. None of the patients had left upper extremity ischemia or paraplegia during the perioperative period. The half-year LSA patency rate was 99.3%, the 1-year LSA patency rate was 97.58%, and the 2-year LSA patency rate was 95.23%.

A Type I endoleak refer to blood entering the false lumen of the vessel and is caused by a stent graft that does not completely seal the primary entry tear of aorta, thus the vessel remains at risk of rupture (34). Therefore, type I endoleak needs to be treated in a timely manner, and it is also one of the main indicators used to evaluate the effect of endoluminal therapy (34).

### Comparison of the clinical data between patients who had Castor stent grafts and hybrid technology and chimney technology and fenestration technology

Hybrid techniques, including carotid-subclavian bypass or subclavian-carotid transposition, have been widely reported and work well overall. Hybrid techniques require temporary clamping of supra-aortic vessels, which involves a risk of cerebral ischemia (35). Bartos reported carotid-axillary bypass grafting as a revascularization method for zone II thoracic aortic endovascular repair, which showed an in-hospital mortality rate of 3% and a patency rate of 97% for the reconstructed bypass, and the perioperative stroke rate was 4% (36). The perioperative complications included brachial plexus stress (1%), sympathetic nerve palsy (1%), wound hematoma (3%), and vertebral artery occlusion in 6% of the patients. During the follow-up period, 3% of the patients developed left upper extremity ischemia and 3% developed subclavian steal syndrome. Other studies have reported complications such as lymphatic leakage, vocal cord paralysis, and even paraplegia after the hybrid technique has been performed (37).

Regarding the chimney technique for the treatment of aortic arch disease, the meta-analysis reported by Ahmad (38) showed that the technical success rate was 91.3%, the perioperative type Ia endoleak rate was 7%, the early type Ia endoleak rate was 9.4%, the incidence of retrograde type A dissection was 1.8%, the 30-day mortality rate was 7.9%, the reintervention rate was 10.6%, the stroke rate was 2.6%, the early target vessel patency rate was 97.9%, and the late target vessel patency rate was 92.9%. In the chimney technique, the two stents are placed running parallel in the aortic arch, and there is an increased risk of endoleak and a gradual decrease in the long-term patency of the target vessel. However, the chimney technique is relatively simple; it does not take a long time to customize the stent, and it is suitable for use in emergency surgery.

For the *in situ* fenestration technique, the results of systematic reviews (39) showed that the overall technical success rate, perioperative mortality and stroke rates were 88.3, 5.9, and 9.5%, respectively, and the perioperative complication rate included 1 case (1%) of retrograde type A aortic dissection, 2 (2%) type II endoleaks, and 3 (3%) strokes. The study included an insufficient number of patients, leading to possible data bias. The fenestration technique will destroy the original overall structure of the stent, resulting in deformation and displacement of the stent, with serious consequences such as branch artery occlusion.

The results of this meta-analysis show that, compared with chimney technology, fenestration technology, and hybrid technology, Castor stent graft technology has the advantages of a higher technical success rate, a lower mortality rate, a lower endoleak rate, and a higher long-term patency rate of the LSA. Studies have shown that the incidence of spinal cord ischemia after TEVAR is 11%, and half of these cases are permanent spinal cord ischemia (40). It is worth noting that there is no report of paraplegia caused by spinal cord ischemia in the studies collected in this meta-analysis, which fully shows that branch stent technology can better protect the patency of the vertebral artery. Especially for patients with right vertebral artery absence, it is more important to protect the left vertebral artery.

### Comparison of Castor stent grafts with other single branch stents

The single-branch stents that are currently used for reconstruction of the LSA include Castor stent grafts (Microport Medical Co., Ltd., Shanghai, China), WeFlow-Tbranch (Weiqiang Medical Co., Ltd., Hangzhou, China), Valiant Mona LSA (Medtronic, Santa Rosa, CA, USA), TAG (Gore, USA) and Nexus (Endospan, Israel). Among them, Castor is a one-piece valgus single-branch design, WeFlow-Tbranch and TAG are modular embedded branch designs, Valiant and Nexus are modular valgus branch designs, and Nexus combined with neck bypass surgery is mainly used for reconstruction of the innominate artery.

In 2015, it was reported that the technical success rate of nine patients who were initially enrolled in the Valiant stent clinical trial was 100%, and as of 2019, there were no patient deaths, left upper extremity ischemia, hemiplegia, aneurysm rupture or conversion to surgery-related events (41, 42). The study included 44 patients again in 2018 for an efficacy study, and the patients will be followed up for 5 years. The results of the follow-up have not yet been published. The branch stent in the TAG stent has a tapered design to improve the closure of the branch stent. In the initial feasibility trial, 22 patients were free of perioperative mortality and stroke complications, but 1 patient had a stent-related death at 6 months of follow-up (43). In 2021, Dake published preliminary results of a TAG single-branch stented vessel, which included 31 patients with a 100% technical success rate. At the 30-day follow-up, the branch vessel patency rate was 100%, the endoleak-free rate was 96.7%, and there were no deaths or hemiplegia (44). During the 1-year follow-up, there were 5 non-stent-related deaths and 1 reintervention, without conversion to surgery. The Nexus Stented Vessel (45) is used for endovascular therapy to reconstruct the innominate artery but requires bypass surgery combining the LCCA and LSA. Among the 28 patients currently treated with Nexus, Nexus has been successfully placed, with a 30-day mortality rate of 7.1% and a stroke rate of 3.6%. The 1-year follow-up results showed an overall case fatality and stroke rate of 17.8% and a stent-related reintervention rate of 10.7%. Compared with the Castor stent graft, research on the above three stents has progressed very slowly, and the clinical efficacy still needs to be confirmed by more comprehensive data.

### Advantages of Castor stent grafts

(I) Single-branch stent technology can seal the dissection opening while retaining the LSA under the premise of an insufficient proximal anchoring length, which can significantly reduce serious complications such as nervous system and upper limb ischemia caused by LSA closure.
(II) The single-branch aortic stent-graft only needs to be introduced and released at one time, the operation is relatively simple, and there is no need to mix this technique with other operations. For some patients at risk of progression of dissection, emergency surgery can be performed within 72 h of the onset, which can avoid retrograde type A dissection and facilitate timely and effective treatment.
(III) The integrated design of Castor stent grafts corresponds better with the physiological and anatomical characteristics of human large blood vessels regarding shape. The direct connection between the branch stent and the main stent improves the stability of the stent structure, which leads to a lower incidence of long-term stent displacement (46).
(IV) The stent-graft is covered by a soft inner sheath as it enters the arch, reducing the risk of both intimal injury and cerebral embolism. The branch portion of the stent-graft is folded by a “cap” made of the same fabric as the soft inner sleeve. This design also prevents intimal damage during the pulling of the branch profile into the branch artery (5).

### Limitations of the Castor stent graft

(I) Although the Castor stent graft and its delivery system have standard operating procedures, the procedure is more complicated than that of the straight-tube stent. In actual operations, there are various problems, such as inaccurate alignment of the branch stent and winding of the guide wire (47).
(II) Compared with the chimney technique, the Castor stent graft technique is more complex because the stent must be customized, which is expensive and is not suitable for emergency surgery but is more suitable for elective surgery (47).
(III) The complex anatomical condition of the aortic arch is not conducive to the precise positioning of the substent on the aortic arch, and local stenosis of the branch will increase the risk of late branch occlusion. Second, the complexity of the procedure also increases the risk of stroke, and the offset in the longitudinal section increases the risk of endoleak, mainly due to torsion of the substent and insufficient apposition of the main stent to the aorta. At present, the substent and the main stent are sutured perpendicular to each other, and the angle between the LSA and the aorta gradually decreases with age, which is more prominent in the type III aortic arch, increasing the risk of branch breakage and endoleak. The substent is released by pulling, which increases the pressure on the aortic wall above the stent body, which is originally the most stressed, and increases the risk of iatrogenic complications.
(IV) For complex aortic lesions involving the LCCA and innominate arteries, CSG still has limitations, which need to be solved by combining the hybrid technique, chimney, or fenestration technique.

### Publication bias assessment and subgroup analysis

**FIGURE 5:**
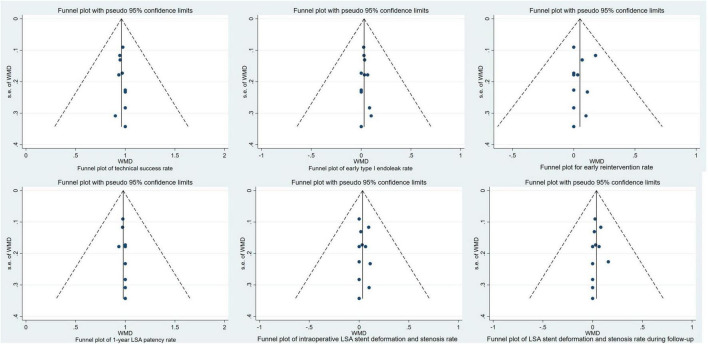
Funnel plots.

In this study, all patients underwent the CSG technique to reconstruct the LSA, and the success rate of LSA preservation was 100%. Among them, five cases of type I endoleak occurred, and the factors that led to the endoleak were too large an angle of the substent, serious distortion of the branch, and poor fit between the stent and the blood vessel. At the same time, it can be found from the funnel plot that the included studies were all concentrated in the midline, and Egger’s test was used to obtain *P* = 0.795 > 0.05, indicating that there was no obvious publication bias in the technical success rate. The funnel plots are shown in Figure 5.

The subgroup analysis of the LSA occlusion stenosis rate during the follow-up period was performed according to the length of follow-up. Group A was followed for more than 12 months, and group B was followed for less than 12 months. The results of the subgroup analysis showed no significant difference between the two subgroups, indicating that the LSA occlusion stenosis rate was minimally associated with the duration of follow-up, and also reflected the stability of the stent. The subgroup analysis forest plot is shown in Supplementary Figure 2.

The main limitation of this systematic review is that these studies were mostly retrospective or observational studies with relatively small sample sizes. Secondly, factors such as different medical centers, different experienced operators, different types of aortic arch lesions, and large differences in the follow-up times between the studies have an impact on the final results. The short-term and mid-term results are only reported in this study, and long-term results from larger and higher-quality studies are still needed to demonstrate the long-term efficacy of the Castor stent graft. In addition, it is important to note that the quality of evidence for all outcomes is between low and very low, so the effect of this intervention needs to be validated by more high-quality studies.

## Conclusion

Finally, through this meta-analysis, we found that Castor stent graft technology has good short- and mid-term clinical efficacy and is an effective treatment for type B aortic dissection with an insufficient proximal anchoring zone. This shows that branch stent technology has great development potential, and its long-term efficacy still needs further clinical observation.

## Data availability statement

The original contributions presented in this study are included in the article/Supplementary material, further inquiries can be directed to the corresponding author.

## Author contributions

SY drafted the manuscript. SY and XC performed the statistical analysis. SY, XC, and YL drafted the figures and legend. SY, XC, GD, DL, GQ, RQ, XS, and QZ designed the outline of the topic and helped on revising the manuscript. All authors contributed to the article and approved the submitted version.

## Footnotes

1
http://www.crd.york.ac.uk/PROSPERO
