# Supplementary figures and images for: Systematic review and meta-analysis of type B aortic dissection involving the left subclavian artery with a Castor stent graft
Source: Front Cardiovasc Med. 2022 Nov 29;9:1052094. doi: 10.3389/fcvm.2022.1052094 (PMC9745178; doi:10.3389/fcvm.2022.1052094)

## Slide 1
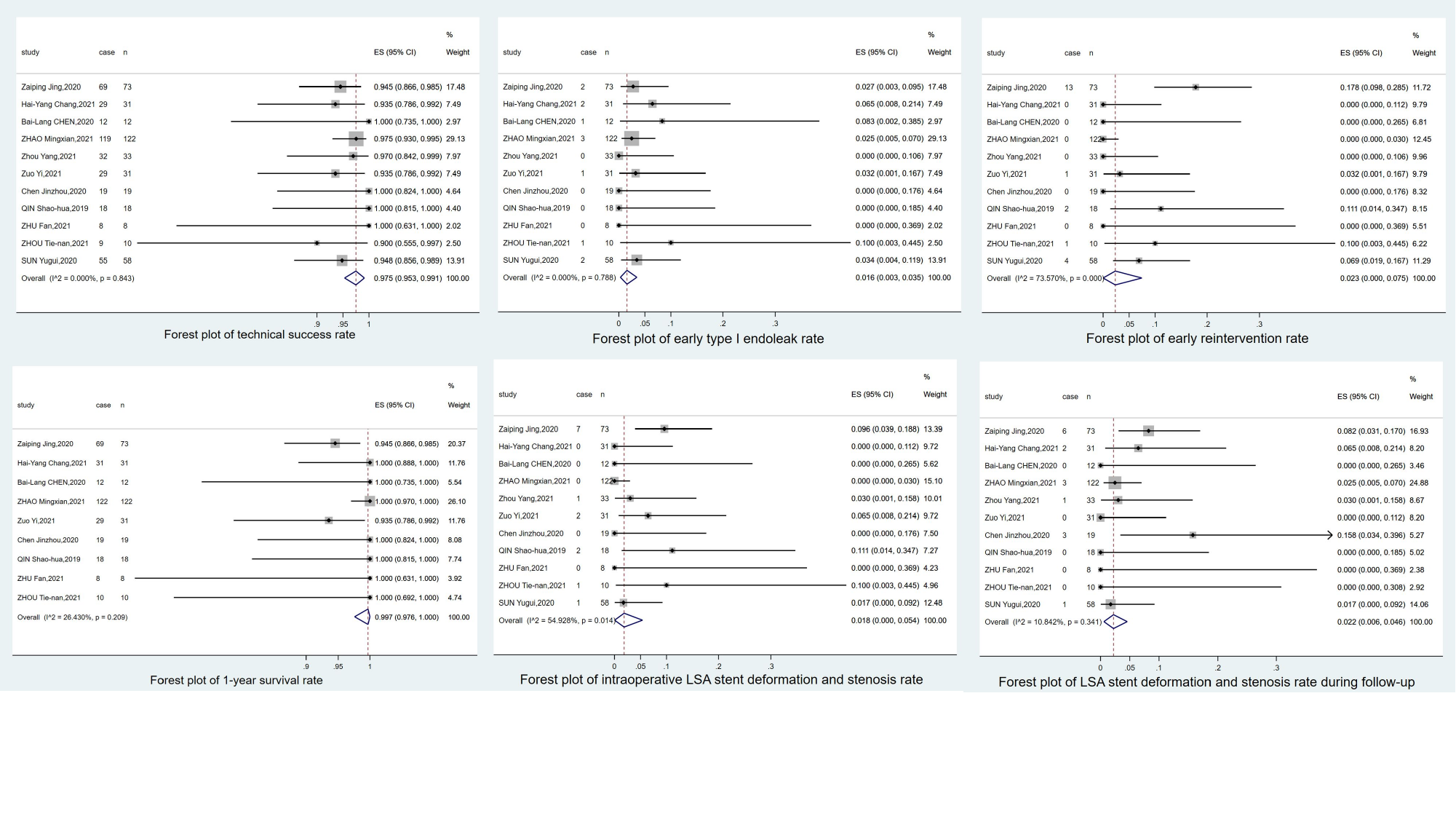

## Slide 2
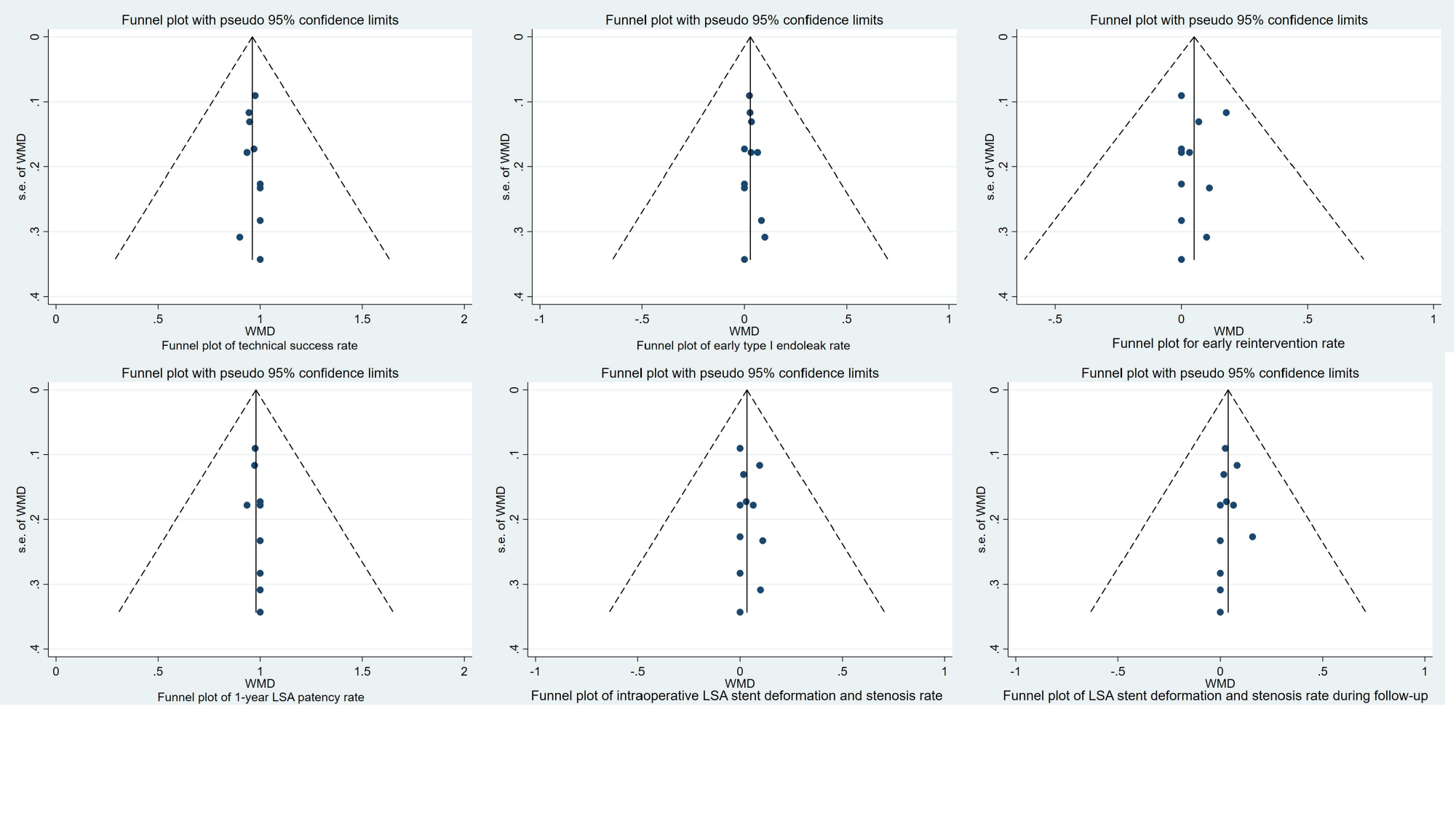

Supplement: Supplementary file 1 [file Presentation_1.PPTX]

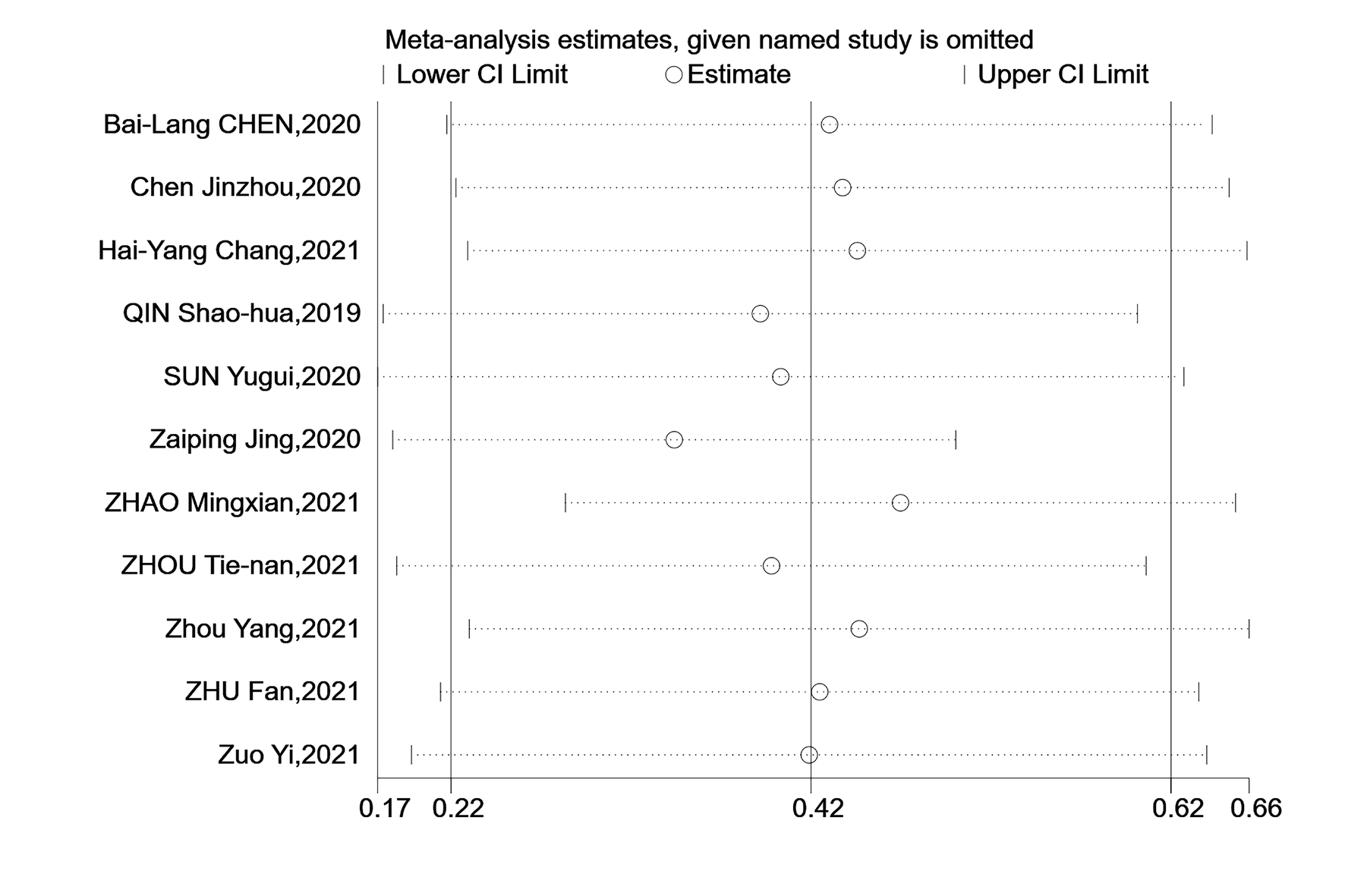

Supplement: Supplementary file 3 [file Image_1.tif]

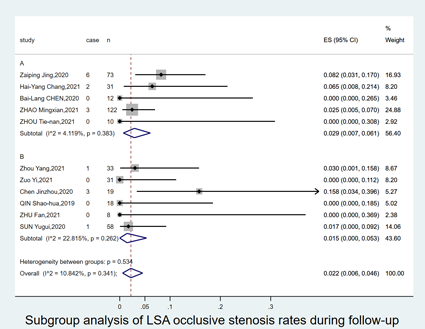

Supplement: Supplementary file 4 [file Image_2.tif]
