# Supplementary material for: Systematic review and meta-analysis of type B aortic dissection involving the left subclavian artery with a Castor stent graft
Source: Front Cardiovasc Med. 2022 Nov 29;9:1052094. doi: 10.3389/fcvm.2022.1052094 (PMC9745178; doi:10.3389/fcvm.2022.1052094)
Supplement: Supplementary file 2 [file Data_Sheet_1.docx]

Supplementary Table S1. Excluded literature and reasons for exclusion

| No. | literature | Author | Year | Journal | Reasons for exclusion |
| --- | --- | --- | --- | --- | --- |
| 1 | Early Outcomes of Left Subclavian Artery Revascularization Using Castor Single-Branched Stent-Graft in the Treatment of Type B Aortic Dissection or Intramural Hematoma | Changcun Fang | 2021 | Annals of Thoracic and Cardiovascular Surgery | Duplication of data with "Hai-Yang Chang, 2021" |
| 2 | Branch Stent-grafting for Endovascular Repair of Chronic Aortic Arch Dissection | Lei Zhang | 2019 | The Journal of Thoracic and Cardiovascular Surgery | Duplication of data with "Zaiping Jing,2020" |
| 3 | Thoracic Endovascular Aortic Repair Combined with Assistant Techniques and Devices for the Treatment of Acute Complicated Stanford Type B Aortic Dissections Involving Aortic Arch | [Tianhua Zhang](https://pubmed.ncbi.nlm.nih.gov/?size=100&term=Zhang+T&cauthor_id=26806251) | 2015 | Annals of Vascular Surgery | Duplication of data with "Zaiping Jing,2020" |
| 4 | Comparison of Chimney Technique and Single-Branched Stent Graft for Treating Patients with Type B Aortic Dissections that Involved the Left Subclavian Artery | [Honggang Zhang](https://pubmed.ncbi.nlm.nih.gov/?size=100&term=Zhang+H&cauthor_id=30564883) | 2018 | Cardiovascular and Interventional Radiology | Duplication of data with "Zaiping Jing,2020" |
| 5 | Outcomes of thoracic endovascular aortic repair for penetrating aortic ulcers involving the left subclavian artery with the Castor single-branched stent graft | Ben Huang | 2022 | European Journal of Cardio-Thoracic Surgery | Duplication of data with "Zaiping Jing,2020" |
| 6 | Implantation of Unibody Single-Branched Stent Graft for Patients with Type B Aortic Dissections Involving the Left Subclavian Artery: 1-Year Follow-Up Outcomes | He Huang | 2017 | Cardiovascular and Interventional Radiology | Duplication of data with "Zaiping Jing,2020" |
| 7 | Endovascular Aortic Repair With Castor Single-Branched Stent-Graft in Treatment of Acute Type B Aortic Syndrome and Aberrant Right Subclavian Artery | Xinyan Pang | 2021 | Vascular and Endovascular Surgery | The study was of the right subclavian artery, not the left clavicular artery, and was not associated with aortic dissection. |
| 8 | Mid-term Comparison of One-Piece Branched Stent-Graft and Chimney Technique Treating Aortic Arch Pathologies | Mingwei Wu | 2022 | Cardiovascular and Interventional Radiology | The study included aneurysms and data were not separate. |
| 9 | Clinical analysis of castor branch integrated stent in the treatment of aortic dissection and aneurysm | Zhou Pengli | 2021 | Chinese Journal of Radiology | The study included aneurysms and data were not separate. |
| 10 | Study of learning curve analysis and operational tips of Castor branched stent-graft | Guo Songlin | 2021 | Chinese Journal of Vascular Surgery (Electronic Edition) | Lack of data during follow-up. |
| 11 | Study on the Correlation Between Timing of Release of Castor Branch Stent and the Risk of Thrombosis | ZENG wen-feng | 2020 | Heilongjiang Medical Journal | The data is indivisible. |
| 12 | Observation of Single Branch Stent in the Treatment of Stanford Type B Aortic Dissection | WANG Chuangsheng | 2020 | Chinese General Practice | Duplication of data with "CHEN Jinzhou,2020" |
| 13 | Branched aortic stenting for arch aortic diseases | Wang Qiong | 2020 | Chinese Journal of General Surgery | The study included aneurysms and data were not separate. |
| 14 | Short-term results of left subclavian artery resconstruction with branched thoracic endovascular aortic repair in the treatment of complex of aortic arch lesions | Pan Hongrui | 2019 | Chinese Journal of General Surgery | The study included aneurysms and data were not separate. |
| 15 | Clinical research of branched stent-graft in the treatment of Stanford B aortic dissection with insufficient landing zone | Lan Yongrong | 2020 | Journal of Trauma and Emergency(Electronic Version) | The data is indivisible. |
| 16 | Preliminary experience of Castor clinical trial | Jiang weiliang | 2014 | Journal of Chinese Physician | Duplication of data with "Zaiping Jing,2020" |
| 17 | Clinical application of domestic novel branched stent in thoracic aortic disease | SUN Yugui | 2020 | International Journal of Cardiovascular Disease | The data is indivisible and duplication of data with "SUN Yugui,2020" . |
| 18 | Total Thoracic Endovascular Aortic Repair for Lesions Involving Aortic Arch：Mid-to Long-term Results of 95 Cases | Li Qingle | 2020 | Chinese Journal of Minimally Invasive Surgery | Duplication of data with "Hai-Yang Chang, 2021" |
| 19 | Endovascular repair of complex aortic arch lesion with application of recanalize left subclaivian artery in intracavitary | Xuan Haiyang | 2021 | Chinese Journal of Thoracic and Cardiovascular Surgery | Duplication of data with "Zuo Yi, 2021" |
| 20 | Clinical application of single-branch Castor stents under total puncture in the treatment of aortic dissection. | Zhang Siyuan | 2020 | Electronic Journal of Clinical Medical Literature | The data is indivisible. |
| 21 | Endovascular repair of complex aortic arch lesion with single branched stent graft | MENG Weixin | 2019 | Journal of Cardiovascular and Pulmonary Diseases | The study included aneurysms and data were not separate. |
| 22 | Analysis of the clinical efficacy of unibody single-branched stent graft implantation for patients with thoracic aortic disease | Xu Yingding | 2022 | Chinese Journal of Vascular Surgery (Electronic Edition) | The study included aortic aneurysms and aortic ulcers, and the data were not separated. |

Supplementary Table S2. Follow-up outcomes

| **Reference** | **Leakage during follow-up** | **stroke** | **paraplegia** | **retrograde type A dissection** | **survival rate** | | |
| --- | --- | --- | --- | --- | --- | --- | --- |
|  |  |  |  |  | **3 months** | **6 months** | **1 year** |
| Zaiping Jing,2020 | 0 | 0 | 0 | 0 | 95.9% | 95.9% | 94.5% |
| Hai-Yang Chang,2021 | 0 | 0 | 0 | 0 | 100% | 100% | 100% |
| Bai-Lang CHEN,2020 | 1 | 0 | 0 | 0 | 100% | 100% | 100% |
| ZHAO Mingxian,2021 | 3 | 1 | 0 | 1 | 100% | 100% | 100% |
| Zhou Yang,2021 | 0 | 0 | 0 | 0 | NA | NA | NA |
| Zuo Yi,2021 | 1 | 0 | 0 | 0 | 93.5% | 93.5% | 93.5% |
| Chen Jinzhou,2020 | 0 | 0 | 0 | 0 | 100% | 100% | 100% |
| QIN Shao-hua,2019 | 0 | 0 | 0 | 1 | 100% | 100% | 100% |
| ZHU Fan,2021 | 0 | 0 | 0 | 0 | 100% | 100% | 100% |
| ZHOU Tie-nan,2021 | 1 | NA | NA | NA | 100% | 100% | 100% |
| SUN Yugui,2020 | NA | NA | NA | NA | NA | NA | NA |

Supplementary Table S3. Result of GRADE Assessment

| **Outcomes** | **№ of participants (studies) Follow-up** | **Certainty of the evidence (GRADE)** | **Relative effect (95% CI)** |
| --- | --- | --- | --- |
|  |  |  |  |
| Technical success rate | 415 (11 observational studies) | ⨁⨁◯◯ Low^a^ | **0.975** 0.963 to 0.991 |
| Early type I endoleak rate | 415 (11 observational studies) | ⨁◯◯◯ Very low^a^ | **0.016**  (0.003 to 0.035) |
| Perioperative stroke rate | 415 (11 observational studies) | ⨁◯◯◯ Very low^a^ | **0.000**  (0.000 to 0.005) |
| Early reintervention rate | 342 (10 observational studies) | ⨁◯◯◯ Very low^a^ | **0.009**  (0.00 to 0.04) |
| One year survival rate | 415 (11 observational studies) | ⨁⨁◯◯ Low^a^ | **0.997**  (0.976 to 1.000) |
| Intraoperative LSA stent deformation and stenosis rate | 415 (11 observational studies) | ⨁◯◯◯ Very low^a^ | **0.018**  (0.000 to 0.054) |
| Follow-up LSA stent deformation and stenosis rate | 415 (11 observational studies) | ⨁◯◯◯ Very low^a,b^ | **0.022**  (0.006 to 0.046) |
| Follow-up reintervention rate | 415 (11 observational studies) | ⨁◯◯◯ Very low^a,b^ | **0.15**  (0.000 to 0.055) |

a. Small sample size; b. Secondary outcome.
